# Rad9, a 53BP1 Ortholog of Budding Yeast, Is Insensitive to Spo11-Induced Double-Strand Breaks During Meiosis

**DOI:** 10.3389/fcell.2021.635383

**Published:** 2021-03-25

**Authors:** Takehiko Usui, Akira Shinohara

**Affiliations:** Institute for Protein Research, Osaka University, Suita, Japan

**Keywords:** Rad9/53BP1, Rad53, DDR (DNA damage response), checkpoint, meiosis, recombination

## Abstract

Exogenous double-strand breaks (DSBs) induce a DNA damage response during mitosis as well as meiosis. The DNA damage response is mediated by a cascade involving Mec1/Tel1 (ATR/ATM) and Rad53 (Chk2) kinases. Meiotic cells are programmed to form DSBs for the initiation of meiotic recombination. In budding yeast, Spo11-mediated meiotic DSBs activate Mec1/Tel1, but not Rad53; however, the mechanism underlying the insensitivity of Rad53 to meiotic DSBs remains largely unknown. In this study, we found that meiotic cells activate Rad53 in response to exogenous DSBs and that this activation is dependent on an epigenetic marker, Dot1-dependent histone H3K79 methylation, which becomes a scaffold of an Rad53 mediator, Rad9, an ortholog of 53BP1. In contrast, Rad9 is insensitive to meiotic programmed DSBs. This insensitiveness of Rad9 derives from its inability to bind to the DSBs. Indeed, artificial tethering of Rad9 to the meiotic DSBs activated Rad53. The artificial activation of Rad53 kinase in meiosis decreases the repair of meiotic DSBs. These results suggest that the suppression of Rad53 activation is a key event in initiating a meiotic program that repairs programmed DSBs.

## Introduction

DNA double-strand breaks (DSBs) are accidentally formed in cells with endogenous and exogenous DNA damage. DSBs induce a DNA damage response enabling the cell to cope with the damage and to coordinate DNA damage tolerance, including the repair of DSBs, throughout progression in the cell cycle. Failure of the DNA damage response to DSBs leads to instability of the genome, which is often associated with the onset of cancer.

DNA damage response is a complex biological pathway that is accompanied by a signaling cascade involving multiple kinases. In mitotic dividing cells, DSBs activate 2 key kinases, Mec1 (ATR in humans), a member of the PI3-kinase family, and Tel1 (ATM in humans) [reviewed in Nyberg et al. (2002))]. Mec1 is recruited to single-stranded DNA (ssDNA) in a Ddc2 (ATRIP)-dependent manner. Ddc2 recognizes replication protein-A (RPA)-coated ssDNA (Paciotti et al., 2000; Zou and Elledge, 2003). Tel1 binds to the ends of DSBs through Xrs2/Nbs1 in the Mre11-Rad50-Xrs2/Nbs1 (MRX/N in humans) complex (Nakada et al., 2003). Activated Mec1/Tel1 then stimulates Rad53 kinase (Chk2 in humans) (Sanchez et al., 1996; Sun et al., 1996) and transduces a signal to downstream effector pathways (Allen et al., 1994). Rad9 (53BP1 in humans) is a key modulator of Rad53 activation (Sun et al., 1998; Schwartz et al., 2002). Rad9 is recruited to the DNA damage area using 2 distinct histone modifications, histone H2AS129 phosphorylation (γ-H2AX in humans) and H3K79 methylation (Giannattasio et al., 2005; Wysocki et al., 2005; Toh et al., 2006; Grenon et al., 2007; Hammet et al., 2007). H2AS129 phosphorylation and H3K79 methylation are recognized by 2 domains of Rad9, that is, BRCT and Tudor, respectively. Histone H2AS129 phosphorylation is DNA damage-dependent and is catalyzed by Mec1 and Tel1 (Shroff et al., 2004). On the other hand, H3K79 methylation is constitutive and is mediated by Dot1 methyltransferase. Rad9 recruited to DSBs is phosphorylated by Mec1/Tel1 (Vialard et al., 1998; Schwartz et al., 2002), leading to phosphorylation-dependent oligomerization of Rad9 (Usui et al., 2009). These upstream events promote a scaffold function of Rad9 for Mec1/Tel1-dependent Rad53 activation (Gilbert et al., 2001; Sweeney et al., 2005). Rad9 facilitates Mec1/Tel1 phosphorylation of Rad53 (Sweeney et al., 2005). This *trans*-phosphorylation by Mec1/Tel1 appears to function as a priming event for Rad53 activation. Rad9 also mediates the self-phosphorylation of Rad53 (Gilbert et al., 2001). The *cis*-phosphorylation induces the conversion of Rad53 into a fully activated form (Pellicioli et al., 1999; Gilbert et al., 2001; Sweeney et al., 2005). Moreover, Rad9 undergoes phosphorylation by cyclin-dependent kinase (CDK) to modulate the DNA damage response in a cell-cycle dependent manner (Bonilla et al., 2008; Granata et al., 2010; Pfander and Diffley, 2011). This phosphorylation promotes the binding of Rad9 to the other DDR mediator protein, Dpb11 (Pfander and Diffley, 2011; Puddu et al., 2011; di Cicco et al., 2017), whose recruitment to DSB sites is dependent of the Rad17-Ddc1-Mec3 checkpoint clamp (Rad9-Rad1-Hus1 in other organisms).

Although DSBs are usually an accidental event in cells, several somatic cells also introduce programmed DSBs in their genomes in order to promote cellular differentiation, such as mating type switching in fungi and antigen receptor rearrangement in the immune cells of vertebrates [reviewed in Haber (2012), Alt et al. (2013))]. Moreover, meiotic cells in sexually reproducing eukaryotes develop a program to induce hundreds of DSBs in their genomes (∼160 in the budding yeast, ∼300 in humans/mice, and more than ∼1,000 in the lily), which initiates meiotic recombination (Terasawa et al., 1995; Barlow et al., 1997; Buhler et al., 2007; Kauppi et al., 2011; Pan et al., 2011). Such recombination enables the exchange of paternal and maternal DNA molecules to form a crossover (CO) [reviewed in Bishop and Zickler (2004))]. The CO is converted into an exchange of homologous chromosome axes, which are cytologically visualized as chiasma. The chiasma promotes proper segregation of homologous chromosomes at meiosis I along with cohesion distal to chiasmata. The meiotic recombination is essential for meiosis, and thus gamete formation. Any defects in recombination result in mis-segregation of homologous chromosomes, leading to the formation of aneuploidy gametes.

The formation of meiotic DSBs is catalyzed by Spo11, a meiosis-specific topoisomerase VI-like protein (Bergerat et al., 1997; Keeney et al., 1997). Spo11-induced DSBs are processed to generate a 3′-overhanging ssDNA. This ssDNA is coated with RPA to become a template for homology search by 2 RecA homologs, Rad51 and meiosis-specific Dmc1 (Bishop et al., 1992; Shinohara et al., 1992). After homology search by Rad51 and Dmc1, the ssDNA invades homologous double-stranded DNAs (dsDNAs). The interaction between the ssDNA and dsDNA leads to the formation of a recombination intermediate, single-end invasion (SEI) (Allers and Lichten, 2001b; Hunter and Kleckner, 2001). The SEI is then converted into the next intermediate with double Holliday structures (dHJs) (Schwacha and Kleckner, 1995). The dHJ is predominantly resolved into reciprocal exchange molecules, COs (Allers and Lichten, 2001a; Hunter and Kleckner, 2001; Borner et al., 2004). In meiosis, recombination preferentially occurs between homologous chromosomes rather than between sister chromatids, as seen in mitotic cells (interhomolog bias) (Schwacha and Kleckner, 1997).

Recent studies have shown that canonical DNA damage signaling is non-responsive to ∼160 Spo11-induced DSBs in meiotic cells of the budding yeast (Lydall et al., 1996; Carballo et al., 2008; Cartagena-Lirola et al., 2008). This is quite different from the response of vegetatively growing cells, which are very sensitive even to a single DSB (Lee et al., 1998; Pellicioli et al., 2001). Spo11-induced DSBs are known to activate Mec1/Tel1, which in turn activates a meiosis-specific Rad53 paralog, Mek1/Mre4 kinase in the context of chromatin (Hollingsworth and Gaglione, 2019). Mec1/Tel1-dependent phosphorylation of a Mek1 adaptor protein, Hop1, provides the binding site of Mek1 for oligomerization for the activation (Niu et al., 2005; Carballo et al., 2008). Activated Mek1 promotes interhomolog recombination and CO formation as well as activates the recombination checkpoint during prophase I (Hollingsworth and Gaglione, 2019). Importantly, Spo11-induced DSBs do not activate Rad53 (Carballo et al., 2008; Cartagena-Lirola et al., 2008), suggesting that the upstream activation of the canonical pathway is turned on but that the downstream events are suppressed. Indeed, meiotic cells are not intrinsically inert to Rad53 activation; the treatment of *spo11* mutant cells with exogenous DNA damaging agents, such as phleomycin, stimulates Rad53 kinase activity (Cartagena-Lirola et al., 2008). Moreover, the artificial targeting of Rad53, through fusion to Ddc2 (Lee et al., 2004), to ssDNAs of Spo11-induced DSBs induces Rad53 activation in meiosis (Cartagena-Lirola et al., 2008). Previous study also showed that over-expression of Rad53 during meiosis activates Rad53 kinase activity (Usui and Kanehara, 2013). Therefore, it is postulated that meiotic cells develop a mechanism to make Rad53 insensitive to Spo11-dependent DSBs. The mechanism of this insensitiveness of Rad53 to meiotic DSBs remains unknown.

In this study, we showed that, compared to mitotic cells, meiotic cells rely more on Dot1-dependent histone H3K79 methylation to activate Rad53 in response to exogenous DSBs. Moreover, like Rad53 tethering (Cartagena-Lirola et al., 2008), the artificial tethering of Rad9 to meiotic Spo11-mediated DSBs by fusion with Ddc2 stimulated Rad53 kinase activity. Consistent with this, a chromatin-immunoprecipitation (ChIP) assay revealed minimal binding of Rad9 to meiotic DSBs. These results suggest that meiotic DSBs are masked to Rad9 in order to downregulate Rad53 kinase activity in meiotic prophase I. Untimely activation of Rad53 in meiotic prophase I impairs chromosome events such as meiotic recombination. We propose that meiotic cells execute a program that suppresses a part of the DNA damage response to develop meiosis-specific DSB-induced chromosome metabolism.

## Materials and Methods

### Strains and Plasmids

All the strains described here, with the exception of HO endonuclease-inducible strains, are derivatives of SK1 diploids. The strain list is provided in Supplementary Table 1. The pUS48 plasmid harbored *DDC2-3xHA-RAD9* fused with a 0.8-kb DNA fragment containing the *DMC1* 5′-UTR fragment followed by the *kan* gene from a pFA6a-KANMX6 plasmid (Longtine et al., 1998). Like the pUS48, the pUS49 plasmid harbored *3xHA-RAD9* fused with the DNA fragment containing the *DMC1* 5′-UTR fragment followed by the *kamMX6* gene. To integrate *DMC1p-DDC2-3xHA-RAD9* or -*3xHA-RAD9* into the yeast genome, SK1 wild-type cells were transformed with *Eco*RI-digested pUS48 or pUS49 and were grown on YPAD with 100 μg/mL of G418. The plasmids pUS50, pUS72, pUS73, and pUS70 were used to prepare *DDC2*-fusion strains carrying *rad9-7A* (*T390A T398A T410A T427A S435A T457A T603A*), *rad9-Y798A*, and *rad9-K1088M* mutations and a genomic *rad9-Y798A* mutant, respectively. Yeast strains carrying N-terminal 3xHA-tagged *RAD9* (Usui et al., 2009), C-terminal 6xFLAG-tagged *RAD53* (Usui and Petrini, 2007), *rad53-KD* (*K227A*) (Usui and Petrini, 2007), *hht1-K79R*, and *hht2-K79R* were prepared by the 2-step integration method (Kaiser et al., 1994) using the pRS406-based plasmids, pRS406-HA-RAD9, pTAP12, pTAP10, pUS52, and pUS53, respectively. Deletion mutants of the *DMC1*, *DOT1*, *RAD9*, and *SPO11* genes and a *DDC2-3xHA* strain were constructed using a polymerase chain reaction (PCR)-based method (Longtine et al., 1998). The sequences of primers used in the experiments are shown in Supplementary Table 2.

### Antibodies

Anti-Flag (M2 Sigma or Wako), anti-HA (12CA5 for western blot, 16B12 for immunostaining, and F-7 for ChIP), anti-α-tubulin (Serotec), rabbit anti-Rad51 (Shinohara et al., 1992), anti-Dmc1 (Hayase et al., 2004), anti-Rad9 (Usui and Petrini, 2007), anti-Zip1 (Shinohara et al., 2008), and anti-H3K79-3me (Abcam) were used for western blot and immunostaining.

### Meiotic Cell Analyses and DNA Damage Treatment

After SK1 diploid cells entered into meiosis, meiotic cell cycle progression was monitored by 4′,6-Diamidino-2-phenylindole (DAPI) staining as described previously (Hayase et al., 2004). The DNA content of fixed meiotic cells was examined with a FACS Calibur flow cytometer (BD Biosciences) after staining with propidium iodide. Physical analyses for meiotic DSBs and crossover recombinants at the *HIS4-LEU2* locus were performed as described previously (Storlazzi et al., 1995; Shinohara et al., 1997). To induce accidental DSBs in meiosis, cells were incubated with 5 μg/ml of phleomycin for 30 min at 3.5 h after incubation with sporulation media (SPM).

### Cytological Analysis

Spreads of the meiotic nuclei was prepared as described previously (Bishop, 1994; Shinohara et al., 1997). Immunostained samples were observed as described previously (Shinohara et al., 2000) using an epifluorescent microscope (Olympus BX51 with a 100× objective; NA, 1.4), and images were captured with a CCD camera (Cool Snap, Roper) and processed using iVision (Silicon) and Photoshop (Adobe) applications.

### Western Blotting and *in situ* Autophosphorylation (ISA) Assay

For western blot analysis, trichloroacetic acid (TCA)-precipitated cell extracts were prepared as follows: Meiotic cells (2 × 10^8^) were fixed in 0.5 mL of 20% TCA and centrifuged. The cell pellets were disrupted with glass beads in 0.25 mL of 20% TCA using a bead shocker (5 cycles of 2500 rpm, 60 s ON/OFF, Yasui Kikai). TCA precipitates were collected by centrifugation (4,000 rpm, 3 min) and were suspended in 0.24 mL of the sodium dodecyl sulfate-polyacrylamide gel electrophoresis (SDS-PAGE) loading buffer supplemented with 0.33 M Tris-HCl (pH 8.0). The ISA assay was performed as described previously (Pellicioli et al., 1999). Briefly, TCA-precipitated cell extracts were fractionated by SDS-PAGE and transferred to a polyvinyldifluoride (PVDF) membrane (Immobilon P, Millipore). The membrane was sequentially treated by denaturation and renaturation solutions, followed by incubation with ^32^P-γ-ATP. ^32^P-incorporation to Rad53 was visualized and quantified with BAS2000 (Fujifilm).

### *HO* Induction During Mitosis

To induce HO DSB, cells carrying a single unrepairable HO DSB site (Shroff et al., 2004) were arrested at G2/M by nocodazole (15 μg/mL) in YP-lactate. Galactose was added up to 2% to induce HO endonuclease.

### Chromatin Immuno-Precipitation (ChIP) Assay

Meiotic induction was performed as described above. Cells were fixed with 1% formaldehyde for 15 min, and cell extracts were prepared as described previously (Hayase et al., 2004). Cell extracts was prepared using a bead shocker (5 cycles of 2,500 rpm, 60 s ON/OFF, Yasui Kikai). The extract corresponding to 3.6 × 10^8^ cells in pertinent mitotic and meiotic conditions were subjected to immunoprecipitation (IP) using Protein-A coated magnetic beads pre-coated with anti-HA (F-7) or anti-Rad51. DNA precipitated with the immune complex was purified by phenol/chloroform extraction and ethanol precipitation after protease-K treatment and quantified by real-time quantitative PCR (Chromo 4, Bio-Rad) using SyberGreen system (EvaGreen Supermix, Bio-Rad). The ChIP primers used were 5′-GGGTTTATAGTGGTGCCGTTC and 5′-ATGCAACGAAGCTTCCTGGC for *HIS4-LEU2*, 5′-ATGCTGAAGTACGTGGTGACGGAT and 5′-CCTCCGCC ACGACCACACTCT for 0.05 kb from HO-DSB, and 5′-GGTGTGCGGAGTAATCATTTGAGG and 5′-TTATAGGAGA CAGTTTTTCCATCAA for *SMC1*.

## Results

### Histone H3K79 Methylation Is Necessary for Rad9-Dependent Rad53 Activation in Meiotic Cells After Phleomycin Treatment

**FIGURE 1:**
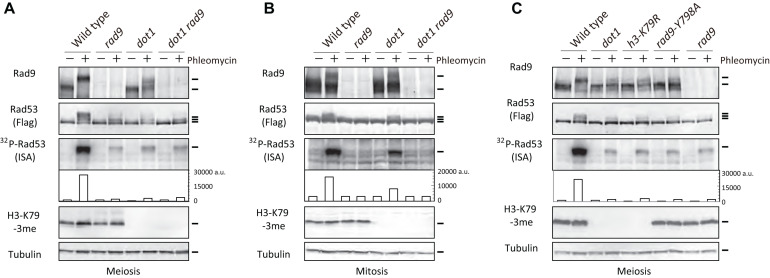
H3K79me is required for Rad9-dependent Rad53 activation in meiosis after phleomycin treatment. **(A)** TCA-precipitated cell extracts were prepared from wild-type (USY543/544), *rad9* (USY524/525), *dot1* (USY526/527), and *dot1 rad9* (USY522/523) diploid strains, and were analyzed by western blotting using anti-Rad9, anti-Flag (for Rad53), anti-H3K79-3me, and anti-tubulin antibodies. “^32^P-Rad53” represented ^32^P-incorporation to Rad53 in the ISA assay. Band intensities were quantified and are shown as arbitrary units without normalization (fourth panels). Cell extracts derived from 4 × 10^6^ cells were loaded for the ISA assay. **(A,C)** Cell extracts were prepared from 3.5-h meiotic cells before (labeled as “–”) and after 30-min treatment with 5 μg/mL of phleomycin (labeled as “+”). **(B)** Diploid cells of the indicated strains were arrested at G2/M by nocodazole and treated with 5 μg/mL of phleomycin for 30 min, followed by TCA precipitation. Wild-type (USY543/544), *rad9* (USY524/525), *dot1* (USY526/527), and *dot1 rad9* (USY522/523) were used in the experiment. Results of an independent experiment are shown in Supplementary Figure 1A. **(C)** Rad53 activation was examined in various strains in meiosis as shown in **(A)**. Wild-type (USY543/544), *rad9* (USY524/525), *dot1* (USY526/527), *h3-K79R* (USY693/677), and *rad9-Y798A* (USY758/759) diploid strains were used. Results of an independent experiment are shown in Supplementary Figure 1B.

Although Rad53 is not activated in response to endogenous Spo11-mediated DNA DSBs, Rad53 in meiotic cells is activated by exogenous DSBs (Cartagena-Lirola et al., 2008). Treatment of meiotic *spo11* mutant cells, which are defective in meiotic DSB formation (Bergerat et al., 1997; Keeney et al., 1997), with a radiomimetic agent, phleomycin, activates Rad53, and this activation depends on Rad9, as seen in vegetatively growing wild-type cells. This indicates that the Rad9-Rad53 axis is functional during meiosis in response to accidental DSBs (Cartagena-Lirola et al., 2008). We confirmed this by treating wild-type meiotic cells with phleomycin. Treatment of meiotic prophase cells grown in sporulation media (SPM) for 3.5 h with phleomycin for 30 min (by 4 h) induced band shifts of Rad53-Flag on western blots (Figure 1A). The band shifts of Rad53 corresponded with multiple phosphorylation of Rad53 accompanied with the activation of its kinase (Pellicioli et al., 1999). Rad53 kinase activity was tested for autophosphorylation activity of Rad53 using an *in situ* autophosphorylation (ISA) assay (Pellicioli et al., 1999). This finding suggests that Spo11-mediated endogenous DSBs do not interfere with the Rad53 activation by exogenous DSBs. Moreover, as shown previously (Cartagena-Lirola et al., 2008), untreated wild-type meiotic cells, which have ∼160 DSBs on their genomes, showed little band shifts or little autophosphorylation of Rad53 (Figure 1A and Supplementary Figure 1A).

As with mitotic cells, the treatment of meiotic wild-type cells with phleomycin leads to Rad53 activation in a Rad9-dependent manner. Meiotic *rad9* deletion cells showed reduced levels of Rad53 autophosphorylation with little band-shifts in response to phleomycin treatment (Figure 1A). This finding is consistent with the observed Rad9 dependency of Rad53 activation in meiotic *spo11* cells with DNA damage (Cartagena-Lirola et al., 2008). Moreover, the treatment of meiotic wild-type cells with phleomycin induced a band shift of Rad9 (Figure 1A and Supplementary Figure 1A), which is likely to be an activated form with multiple phosphorylation.

Methylation of histone H3K79 (H3K79me) by Dot1 methyltransferase is important, but not essential, for the ability of Rad9 to mediate Rad53 activation in mitosis (Giannattasio et al., 2005; Wysocki et al., 2005; Toh et al., 2006). In order to determine the involvement of Dot1 in Rad9-dependent Rad53 activation in meiosis, we analyzed Rad53 activation in meiotic *dot1* cells treated with phleomycin. The *dot1* deletion mutation, which abolished H3K79me, substantially decreased Rad53 activation in response to phleomycin; this decrease in activation was detected by reduced band shifts as well as decreased autophosphorylation levels (Figure 1A and Supplementary Figure 1A). This deficiency of Rad53 activation in the *dot1* mutant was almost identical to that in the *rad9* mutant. Damage-induced Rad9 band shifts were also reduced in the *dot1* mutant. The *dot1 rad9* double mutant showed similar levels of reduced Rad53 activation to those of either single mutant (Figure 1A), suggesting that Dot1 and Rad9 function in the same pathway for phleomycin-dependent Rad53 activation in meiotic cells.

We confirmed that mitotic *dot1* mutant cells are capable of activating Rad53 in response to phleomycin. Previous studies have used only haploid cells, and therefore we used G2/M-arrested diploid cells for treatment with a microtubule polymerization inhibitor, nocodazole. As shown in Figure 1B, wild-type G2/M-arrested diploid cells activated Rad53 in response to phleomycin. In addition, the *rad9* deletion completely abolished the activation. The *dot1* mutant did show some activation of Rad53, but at half the level of that of the wild type, which was determined by the autophosphorylation activity (Figure 1B and Supplementary Figure 1B). Consistent with this result, like wild-type cells, the *dot1* mutant cells exhibited a Rad9 band shift after DNA damage treatment (Figure 1B).

To confirm the role of Dot1 through H3K79 methylation in Rad53 activation during meiosis, we created a histone *H3K79R* mutant strain (*hht1-K79R, hht2-K79R*; *h3-K79R* for simplicity) that was defective in Dot1-dependent methylation (Ontoso et al., 2013, Bani Ismail et al., 2014) (Figure 1C). In this strain, the histone H3 mutant proteins were expressed from the endogenous promoters of 2 copies of the histone H3 genes (*HHT1* and *HHT2*) on the genome. In meiosis, the *h3-K79R* mutant cells showed reduced Rad53 band shifts as well as reduced autophosphorylation when treated with phleomycin (Figure 1C). Decreased levels of activation in the *h3-K79R* mutant were similar to those observed in the absence of *DOT1*. In addition, the band shift of Rad9 was compromised in the *h3-K79R* mutant in a manner similar to that observed in the *dot1* mutant. These results suggest that Dot1-dependent H3K79 methylation is more critical for meiotic cells to activate Rad53 in response to exogenous DNA damage than it is for mitotic cells.

Histone H3K79 methylation is recognized by the Tudor domain of Rad9 (Grenon et al., 2007; Hammet et al., 2007). The *rad9-Y798A* mutation in the Rad9 Tudor domain is known to decrease the interaction between Rad9 Tudor and H3K79me. Therefore, we also investigated the effect of the *rad9-Y798A* mutation on Rad53 activation in meiotic cells. As shown in Figure 1C, *rad9-Y798A* mutant cells showed reduced Rad53 activation, as seen in the *rad9* null mutant. In addition, by itself, the Rad9-Y798A mutant protein showed minimal band shifting. This finding suggests that, in meiotic cells, Rad9 activates Rad53 in response to DNA damage through the Tudor domain of Rad9, which recognizes Dot1-dependent H3K79 methylation. It is important to stress that exogenous DNA damage slightly activated Rad53 even in the absence of Dot1 or Rad9; however, the residual activation was higher than that in the absence of exogenous DNA damage.

Although exogenous DSBs induce Rad53 activation in meiotic cells, Spo11-induced DSBs do not. Importantly, the *rad9*, *dot1*, *h3-K79R*, and *rad9-Y798A* mutants are all proficient in the activation of Mec1/Tel1 checkpoint kinases, which are upstream of Rad53-Rad9, in response to Spo11-DSBs (our unpublished results). These results suggest that meiotic cells show distinct responses to programmed and accidental DSBs with respect to activating Rad53 kinase. Moreover, it is likely that Spo11-dependent DSBs are masked for the activation of Rad53, but not for the activation of Mec1/Tel1.

### The Ddc2-Rad9 Fusion Protein Activates Rad53 in Response to Meiotic DSBs

**FIGURE 2:**
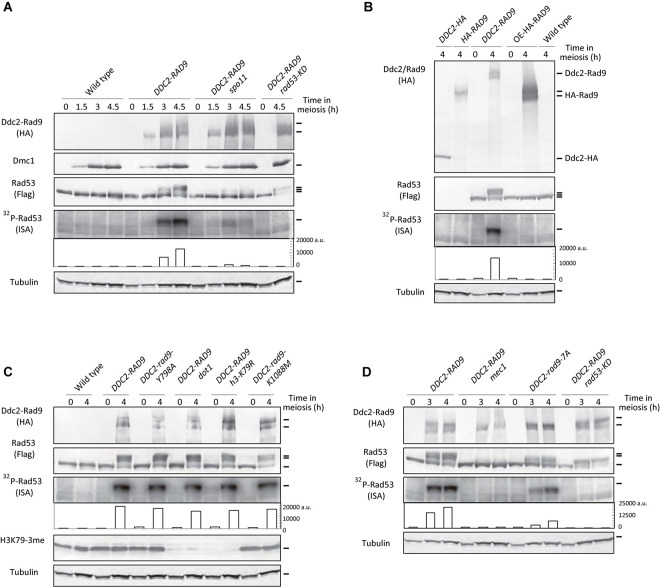
Ddc2-Rad9 fusions activate Rad53 in meiosis. **(A)** TCA-precipitated meiotic cell extracts of the indicated strains were analyzed by western blotting using anti-HA, anti-Flag, anti-H3K79-3me (tri-methylation), anti-Dmc1, and anti–α-tubulin antibodies.^32^P-Rad53 represented ^32^P-incorporation to Rad53 in the ISA assay, and was quantified as shown Figure 1A (fifth panels). Cell extracts derived from 4 × 10^6^ cells were loaded for the ISA assay. Wild-type (USY543/544), *DDC2-RAD9* (USY544/661), *DDC2-RAD9 spo11* (USY783/414), and *DDC2-RAD9 rad53-KD* (USY545/720) were examined. **(B)** Cell extracts were prepared from the strains expressing C-terminal 3x HA-tagged Ddc2 (USY83/84; *DDC2-HA*) and N-terminal 3x HA-tagged Rad9 (USY20/35; *HA-RAD9*) from their native promoters; Ddc2-Rad9 (USY544/671; *DDC2-RAD9*) and 3x HA-tagged Rad9 (USY544/661; *OE-HA-RAD9*) from the *DMC1* promoter; no HA-tagged wild-type strain (USY543/544); wild type. Rad53 was not tagged with Flag in *DDC2-HA* and *HA-RAD9* strains. Flag-Rad53 was expressed from the native promoter. **(C)** Rad53 activation was examined in various strains as in **(A)**. Wild-type (USY543/544), *DDC2-RAD9* (USY544/671), *DDC2-rad9-Y798A* (USY544/776), *DDC2-RAD9 dot1* (USY768/526), *DDC2-RAD9 h3-K79R* (USY770/693), and *DDC2-rad9-K1088M* (USY544/797) were examined. Results of an independent experiment are shown in Supplementary Figure 2C. **(D)** Rad53 activation was examined in various strains as in **(A)**. Wild-type (USY543/544), *DDC2-RAD9* (USY544/671), *DDC2-RAD9 mec1* (USY495/785), *DDC2-rad9-7A* (USY544/667), and *DDC2-RAD9 rad53-KD* (USY4520) were examined. Results of an independent experiment are shown in Supplementary Figure 2D.

Rad53 activation in mitosis is under the control of a multilayered mechanism (Pellicioli and Foiani, 2005). The targeting of Rad9, as well as Rad53, at a DSB site is likely to be crucial for the activation. A previous study showed that tethering Rad53 to meiotic DSBs as a fusion protein to Ddc2 induced Rad53 activation in a Spo11-dependent manner (Cartagena-Lirola et al., 2008). Since Rad9 works upstream of Rad53 in the checkpoint pathway, we questioned whether the tethering of Rad9 to meiotic DSBs might also activate Rad53. To target Rad9 to meiotic DSBs, we placed the Ddc2-3HA-Rad9 fusion protein under the control of a meiosis-specific *DMC1* promoter (referred to as Ddc2-Rad9). The diploid strain (referred to as *DDC2-RAD9*) expressed the Ddc2-Rad9 fusion protein only in meiosis, together with the endogenous (non-tagged) Rad9 protein. Western blots showed that Ddc2-Rad9 was induced in meiotic prophase in a manner similar to the Dmc1 protein (Figure 2A). Ddc2-Rad9 started to appear at 1.5 h and reached a plateau at 3 h. At later periods, Ddc2-Rad9 showed smear bands, suggesting posttranslational modification of Ddc2-Rad9 during meiosis. The expression level of Ddc2-Rad9 from the *DMC1* promoter at 4 h was roughly comparable to that of HA-Rad9 from a native promoter (Figure 2B and Supplementary Figure 4). Concomitant with the expression of Ddc2-Rad9 during meiosis, endogenous Rad53 protein started to show reduced mobility, suggesting the activation of Rad53 (Figure 2A and Supplementary Figure 2). Indeed, the ISA assay confirmed robust autophosphorylation activity of Rad53 after 3-h incubation in meiosis, which was not observed at 0 and 1.5 h. The expression of the Ddc2-Rad9 fusion protein with kinase-dead Rad53, Rad53-KD, abolished the autophosphorylation activity, demonstrating that Rad53 activation by *DDC2-RAD9* was completely dependent on the Rad53 kinase activity. Interestingly, Ddc2-Rad9 induced a smaller, but significant band-shift of Rad53-KD at 4.5 h relative to wild-type Rad53. These results suggest that Ddc2-Rad9 induces Rad53-kinase independent modification of Rad53.

Activation of Rad53 by Ddc2-Rad9 fusion still requires meiotic DSBs. The *spo11* deletion greatly decreased the band shifts as well as the autophosphorylation activity of Rad53 (Figure 2A). Some band shifts of Rad53 were nonetheless apparent, and a low, but increase in Rad53 autophosphorylation was observed, even in the absence of Spo11. This finding indicates that there might be another mechanism for Rad53 activation by Ddc2-Rad9 in a meiotic-DSB independent manner, e.g., in the S phase, since a slight increase in Rad53 autophosphorylation was observed at 1.5 h, the point at which the meiotic S phase occurs.

An increased amount of Rad9 in the *DDC2-RAD9* strain could not explain the activation of Rad53. Expression of non-fusion HA-Rad9 from the *DMC1* promoter was approximately 10-fold higher than that from the endogenous promoter, which did not activate Rad53 (Figure 2B and Supplementary Figure 2B). This result reinforces the idea that the tethering of Rad9 to meiotic DSBs is a key event for inducing Rad53 activation in response to Spo11-induced meiotic DSBs.

We also confirmed the activation of Rad53 kinase activity by Ddc2-Rad9 fusion in the background of the *dmc1*, which is unable to repair meiotic DSBs (Bishop, 1994; Shinohara et al., 2000) and its genetic requirements (see below, with the *spo11*, *mec1*, and *rad53-KD* mutations; Supplementary Figure 3).

### Genetic Requirements for Meiotic Rad53 Activation by Ddc2-Rad9

As shown above, in meiotic cells, exogenous DNA damage-dependent Rad53 activation largely relies on the interaction of Rad9’s Tudor domain with Dot1-dependent H3K79me. Therefore, we questioned whether Rad53 activation by Ddc2-Rad9 in meiosis would also depend on the interaction. Even in the background of *dot1* deletion and *h3-K79R* mutants, Ddc2-Rad9 could still fully activate Rad53 (Figure 2C and Supplementary Figure 2C). The band shifts and autophosphorylation levels of Rad53 in the *dot1* deletion and *h3-K79R* mutants were comparable to those in the wild type. Moreover, consistent with the above results, the introduction of a Tudor mutation, *rad9-Y798A*, in the fusion did not abolish its ability to facilitate the band shifts or autophosphorylation levels of Rad53 (Figure 2C).

In mitosis, in addition to H3K79me, Rad9 localizes to chromatin adjacent to DSBs via the interaction between the BRCT domain of Rad9 and phosphorylation of S129 of histone H2A by Mec1/Tel1 kinases (Hammet et al., 2007). Histone H2AS129 phosphorylation is induced during meiosis in response to Spo11-mediated DSBs (Eichinger and Jentsch, 2010). During meiosis, expression of the Ddc2-fusion of Rad9 with a BRCT domain mutation, *rad9-K1088M*, which impairs the binding of Rad9 to phosphorylated H2AS129 (Hammet et al., 2007), activated Rad53, as in the wild-type fusion protein (Figure 2C). Therefore, these data suggest that Rad53 activation by Ddc2-Rad9 does not require the ability of Rad9 to recognize 2 epigenetic markers that play a critical role in the Rad53 activation to exogenous DSBs in mitotic cells.

In mitosis, the kinase activity of Rad53 largely depends on a major DNA damage kinase, Mec1/ATR (Sanchez et al., 1996; Sun et al., 1996). *DDC2-RAD9*-dependent Rad53 activation in meiosis still requires Mec1. The *mec1 sml1* mutant (the *sml1* mutation suppresses a lethality of the *mec1* deletion) (Zhao et al., 1998) showed little stimulation of Rad53 kinase by *DDC2-RAD9* (Figure 2D). Interestingly, only a few band shifts of Rad53 were observed in the *mec1* mutant, which is in marked contrast to the results for the *DDC2-RAD9 RAD53-KD* strain, which showed a clear band shift with little Rad53 activation (Figure 2A). This finding suggests that Mec1-dependent Rad53 phosphorylation might be an initial key event for Rad53 activation by *DDC2-RAD9*.

Moreover, the Ddc2-Rad9 band shift was compromised in the *mec1* mutant. In the wild-type strain, Ddc2-Rad9 exhibited smear bands, often showing 2 major bands (Figures 2C,D), whereas the *mec1 (sml1)* mutant lacked the major upper band of the Rad9 fusion. Mec1 is known to phosphorylate Rad9 at multiple sites (Schwartz et al., 2002). The *rad9-7A* mutant, with mutations in the major Mec1-phosphorylation sites, T603, and an additional 6 S/T sites of the SQ/TQ cluster domain (SCD), showed decreased Rad53 band shifts and autophosphorylation activity in mitotic cells with exogenous DNA damage (Schwartz et al., 2002). When the Ddc2-Rad9 fusion protein with the *rad9-7A* mutation was expressed in meiosis, the band shift greatly decreased, and the autophosphorylation activity of Rad53 was diminished by ∼1/3 relative to that of the wild-type Ddc2-Rad9 protein at 4 h (Figure 2D and Supplementary Figure 2D). The residual activation of Rad53 might have originated from other, as yet unidentified, phosphorylation sites of Rad9 by Mec1/Tel1. The data suggest that Mec1-mediated phosphorylation of Rad9 remains important for Rad53 activation in *DDC2-RAD9* meiosis.

### The Ddc2-Rad9 Fusion Protein Localizes to Meiotic Chromosomes

**FIGURE 3:**
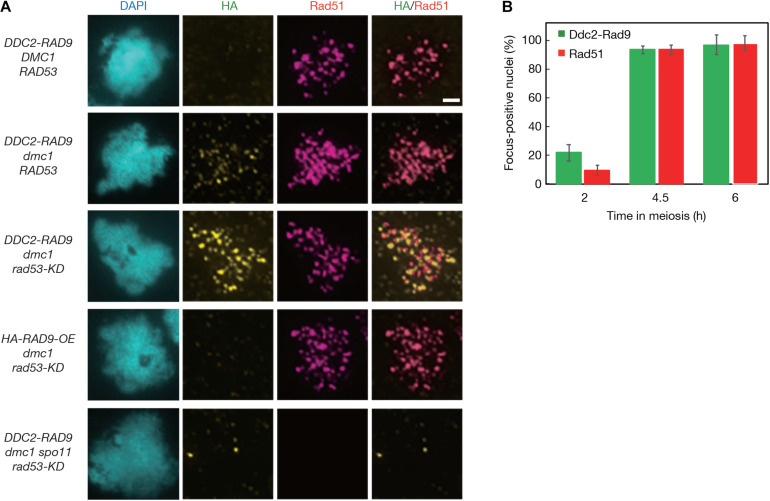
Ddc2-Rad9 localizes to meiotic DSBs. **(A)** The representative images of anti-HA and anti-Rad51 double immunostaining of spread nuclei prepared from *DDC2-RAD9* (USY544/671), *DDC2-RAD9 dmc1 RAD53* (USY580/591), *DDC2-RAD9 dmc1 rad53-KD* (USY582/623), *HA-RAD9-OE dmc1 rad53-KD* (USY582/674), and *DDC2-RAD9 dmc1 spo11 rad53-KD* (USY559/666) strains are shown at 4.5 h in meiosis, except for *DDC2-RAD9 dmc1 RAD53*, which is shown at 6 h in meiosis. Typical DNA images stained with DAPI and merged images by HA- and Rad51-staining are presented. Bar equals 2 μm. **(B)** The percentage of *dmc1*Δ *rad53-KD DDC2-RAD9* nuclei that had more than 5 HA or Rad51 staining foci is presented at the indicated time points. At least 50 nuclei were counted at each time point. Error bars represent standard deviations obtained from 3 independent time courses.

The Ddc2 protein shows punctate staining on meiotic chromosomes when ssDNAs are formed (Refolio et al., 2011). We tried to detect the localization of Ddc2-Rad9 on meiotic chromosomes by immunostaining. However, we did not detect any significant signals for the Ddc2-Rad9 fusion protein on meiotic chromosome spreads in the *DDC2-RAD9* cells by staining with the anti-HA antibody (Figure 3A). The *dmc1* mutant, which cannot repair and accumulate the ssDNA (Bishop et al., 1992), was used for efficient detection of the Ddc2 signal (Refolio et al., 2011). In parallel, staining for Rad51, a marker protein of processed meiotic DSBs (Bishop, 1994), was carried out together with HA-staining. At 6 h in *dmc1* meiosis, when Rad51 foci were formed in more than 94.7 ± 6.8% of nuclei, weak, but significant Ddc2-Rad9 signals as a focus were detectable on chromosomes (Figure 3A and Supplementary Figure 5).

Given that activated Rad53 kinase negatively regulates Rad9 assembly around DSBs (Usui et al., 2009), we further introduced a kinase-dead *rad53-KD* mutation, which may stabilize the Ddc2-Rad9 localization to chromatin. We detected brighter Ddc2-Rad9 foci on chromosomes of the *dmc1* mutant with the *rad53-KD* compared to those in the *dmc1* mutant with wild-type Rad53 (Figure 3A and Supplementary Figure 5). Ddc2-Rad9 localization to chromosomes was specific to meiotic prophase cells. At 0 and 2 h, there was little focus formation of the fusion in the *rad53-KD dmc1* (Figure 3B). Ddc2-Rad9 focus-positive cells appeared concomitantly with Rad51 focus-positive cells (Figure 3B). At 4.5 h, 80% of the cells were positive for Ddc2-Rad9 foci. The average numbers of Ddc2-Rad9 and Rad51 foci in the *rad53-KD dmc1* mutant were 59 ± 5.9 and 71 ± 6.8, respectively (*n* = 18), and 82 ± 4.7% (*n* = 18) of Ddc2-Rad9 was co-localized with Rad51. This supports the idea that Ddc2-Rad9 does indeed bind to ssDNAs for Rad53 activation.

The focus formation of Ddc2-Rad9 in the *dmc1 rad53-KD* mutant, like that of Rad51, once again depended on Spo11, and therefore on DSB formation (Figure 3A). Moreover, simple overexpression of the non-fusion version of HA-Rad9 in the *dmc1 rad53-KD* mutant did not support the localization of the HA-Rad9 protein on meiotic chromosomes (Figure 3A).

### Binding of Rad9 to Meiotic DSBs Is Suppressed

**FIGURE 4:**
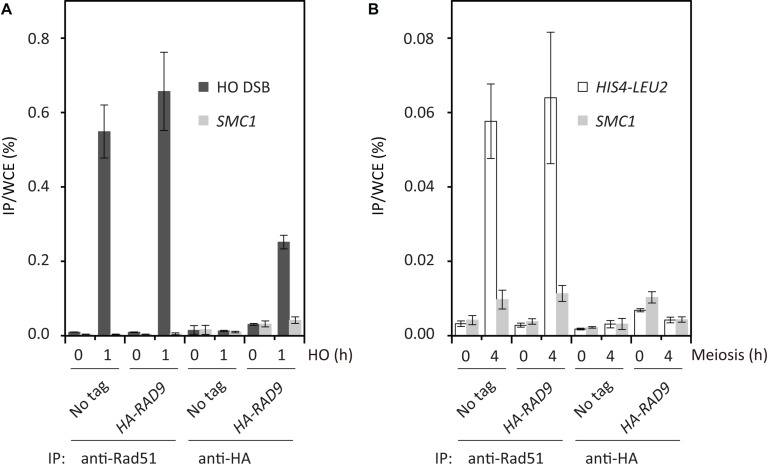
Rad9 is insensitive to meiotic DSBs. Rad9 binding to chromatin adjacent to mitotic HO-induced DSBs **(A)** and to meiotic programmed DSBs at the *HIS4-LEU2* locus **(B)** was tested by ChIP. Formaldehyde-fixed chromatin extracts were prepared at 0 and 1 h after HO DSB induction in G2/M-arrested cells, and at 0 and 4 h after meiotic induction, followed by anti-HA and anti-Rad51 ChIP. Anti-HA- or anti-Rad51-bound DNAs were quantified by real-time PCR. Error bars represent standard deviations obtained from 3 independent cultures. The non-tagged wild-type and *HA-RAD9* strains examined were USY99 and USY100 in **(A)** and NKY1551 and USY2035 in **(B)**, respectively.

The results described above suggested that meiotic cells lack the ability to recruit Rad9 or Rad53 to Spo11-induced DSBs. To confirm this hypothesis, we analyzed the binding of Rad9 to DSBs using a ChIP assay. First, as a control, we carried out ChIP to examine the localization of HA-Rad9 to an HO-endonuclease-induced DSB at the *MAT* locus in vegetative cells (Shroff et al., 2004; Usui et al., 2009). We used a haploid with *GAL1/10-HO*. After 1 h induction of the nuclease in G2/M arrested cells (by nocodazole) with the addition of galactose, increased binding of Rad9 to the DSB was observed at the *MAT* locus, but not at the control locus of *SMC1* (Figure 4A). At the same time, robust binding of Rad51 to the break was detected. Then, we analyzed the binding of HA-Rad9 to a strong meiotic recombination hotspot, the *HIS4-LEU2* locus. While robust binding of Rad51 to the hotspot was detected at 4 h in meiosis, we could detect little binding of Rad9 to the hotspot at 4 h at the same time (Figure 4B). This suggests that Rad9 is insensitive to Spo11-mediated meiotic DSBs, different from the mitotic DSB.

### Ddc2-Rad9 Fusion Delays DSB Repair and Cell Cycle Progression During Meiosis

**FIGURE 5:**
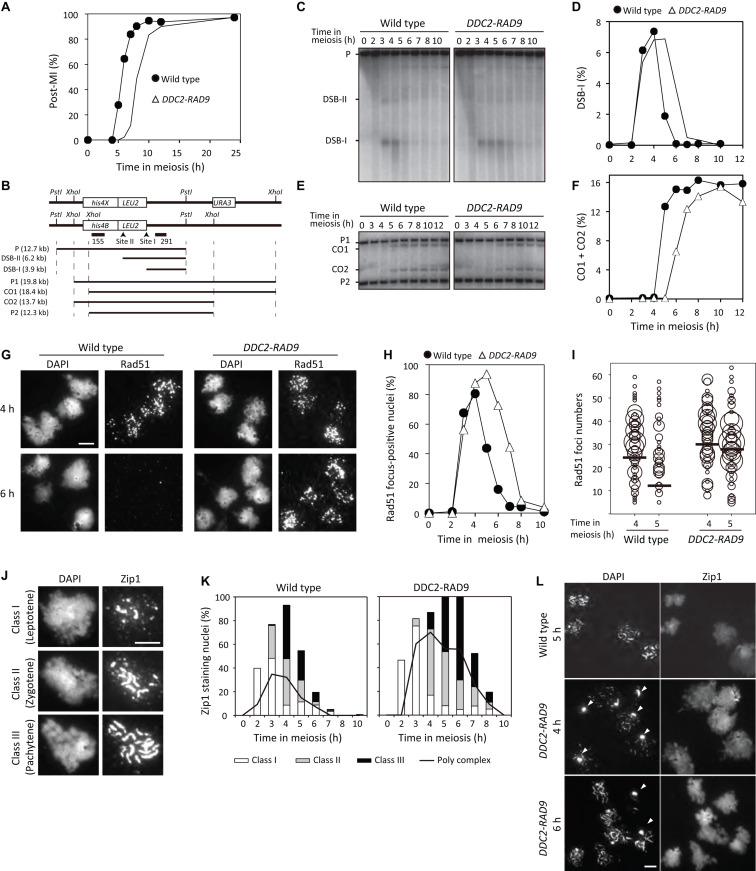
*DDC2-RAD9* impedes cell cycle progression and meiotic recombination. **(A)** Meiotic nuclear divisions were monitored in wild-type (USY543/544) and *DDC2-RAD9* (USY544/671) strains by DAPI staining. The *y*-axis represents the percentage of cells that completed meiosis I and II (Post-MI). At least 200 cells were counted at each time point. **(B)** Schematic presentation of the *HIS4-LEU2* DSB site. **(C,E)** Genomic DNAs were prepared from the indicated strains during the meiotic time course. Meiotic DSBs (DSB-I and DSB-II) **(C)** or crossover recombinants (CO1 and CO2) **(E)** were detected by Southern blot using the probes pNK291 or pNKY155 after genomic DSBs were digested with *Pst*I or *Xho*I, respectively. **(D,F)** Quantitative data of DSB-I **(C)** and CO1 + CO2 **(E)** obtained from Southern blot are plotted. **(G–L)** *DDC2-RAD9* influences localization of Rad51 and Zip1 on meiotic chromosome. **(G)** The representative images of Rad51 immunostaining nuclear foci in spread nuclei prepared from the indicated strains at 4 and 6 h in meiosis are shown. **(H)** The percentage of the indicated cells’ nuclei that had more than 5 Rad51 foci was plotted at the indicated time points. **(I)** Distribution of nuclei according to Rad51 foci number per nucleus at the indicated time is presented using the same data samples as in **(H)**. Only nuclei that had more than 5 Rad51 foci were plotted. The size of the bubbles reflects the percentage of nuclei that had a certain foci number of Rad51 among all nuclei examined. Bars represent the average numbers of Rad51 foci. **(J)** Representative images of Zip1 staining classification are shown. **(K)** Classifications of Zip1 staining at the indicated time in the indicated strains are shown. The line represents the Zip1 polycomplex (PC). At least 100 nuclei were examined at each time point. **(L)** Representative images of Zip1 staining in *DDC2-RAD9* at 4 h and 6 h are shown. Zip1 PCs are indicated by arrowheads. Experiments were performed at least twice, and representative data are shown. Bar equals 5 μm.

We next examined the effect of Ddc2-Rad9 expression, and thus Rad53 activation, on meiosis. The *DDC2-RAD9* diploid showed wild-type spore viability levels (97.9% vs. 98.4% in the wild type; 48 asci). *DDC2-RAD9* cells delayed MI entry by approximately 2 h compared to the wild type (Figure 5A), without any delay in the pre-meiotic S phase (Supplementary Figure 6A).

We investigated meiotic DSB repair and CO formation at the *HIS4-LEU2*, by Southern blotting (Figure 5B) (Cao et al., 1990; Storlazzi et al., 1995). In the wild type, meiotic DSBs formed at 3 h, peaked at 4 h, and then gradually decreased after 5 h (Figures 5C,D). Concomitant with the disappearance of DSBs, COs started to appear at 5 h, and reached a plateau by 6 h in meiosis (Figures 5E,F). *DDC2-RAD9* cells formed DSBs in a manner similar to wild-type cells, but the disappearance of the DSBs was delayed by 1.5 h compared to the wild type (Figures 5C,D). Consistent with the kinetics of DSBs, COs in *DDC2-RAD9* started to appear at 6 h, and reached a plateau by 8 h (Figures 5E,F). Although delayed, the final level of COs in *DDC2-RAD9* cells was comparable to that in the wild type. These data suggest that meiotic DSB repair is delayed in *DDC2-RAD9* meiosis.

To confirm delayed DSB repair in *DDC2-RAD9* meiosis, we observed focus formation of Rad51 on meiotic chromosomes by immunostaining (Bishop, 1994; Shinohara et al., 2000). In the wild type, Rad51 focus-positive nuclei started to appear at 3 h in meiosis, reached a peak at 4 h, and disappeared gradually during further incubation with kinetics similar to those of meiotic DSBs (Figures 5G,H). Rad51 focus-positive nuclei in *DDC2-RAD9* appeared at 3 h and peaked at 5 h. However, the fusion induced a 2-h delay in the disappearance of Rad51 foci (Figures 5G,H). These data support the delay in DSB repair, particularly after Rad51 loading, in the *DDC2-RAD9* strain. Consistent with delayed repair, the average number of Rad51 foci in *DDC2-RAD9* was 30 ± 16 at 4 h (per total nucleus; *n* = 112; Figure 5I), which was higher than that observed in the wild type (24 ± 16, *n* = 113; *P* = 0.009, Mann–Whitney *U* test). Dmc1-focus kinetics were similar to those of the Rad51 focus in *DDC2-RAD9* meiosis (Supplementary Figure 6B). These data demonstrate that Ddc2-Rad9 expression impedes a recombination step after the loading of Rad51/Dmc1. The delayed DSB repair during meiosis of *DDC2-RAD9* cells is consistent with the result that the over-expression of Rad53 delays meiotic DSB repair (Usui and Kanehara, 2013).

Synaptonemal complex (SC) formation was also investigated in the *DDC2-RAD9* strain. We observed the localization of the Zip1 protein, which is the central component of the SC (Sym et al., 1993). As shown in Figures 5J,K, in the wild type, dotty staining was initially observed for Zip1 (class I, leptonema), and the nuclei containing a mixture of dotty and short linear localizations of Zip1 increased (class II, zygonema). Finally, Zip1 was fully extended along chromosomes, which is indicative of a matured SC and complete synapsis (class III, pachynema), and then disappeared subsequently. In *DDC2-RAD9* cells, dotty-staining of Zip1 appeared at 2 h, similar to the wild type (Figure 5K). However, the appearance of short and long linear localization of Zip1 was delayed in *DDC2-RAD9* (Figures 5K,L). At 6 h, *DDC2-RAD9* showed a maximum fraction of Zip1-long lines, whereas the wild type showed the maximum at 4 h. Disassembly of Zip1 lines in the *DDC2-RAD9* strain was delayed by 2 h relative to the wild type. These results suggest that SC elongation is partially defective in *DDC2-RAD9.* Consistent with this, *DDC2-RAD9* cells showed an increased fraction of aggregates (polycomplex) of Zip1 (Figures 5K,L). These results indicate that Ddc2-Rad9 has negative impacts on SC assembly, probably due to delayed DSB repair.

## Discussion

Rad53 is the central effector kinase in the DNA damage checkpoint in mitosis but is not activated during meiosis (Cartagena-Lirola et al., 2008), even in the presence of ∼160 DSBs induced by Spo11 (Buhler et al., 2007; Pan et al., 2011), suggesting that Rad53 activation is masked in response to meiotic programmed DSBs. However, the molecular mechanism underlying the masking of Rad53 is largely unknown. Previous study showed that over-expression of Rad53 during meiosis delays DSB repair, thus the onset of meiosis I, suggesting the presence of silencing mechanism of Rad53 in meiotic cells (Usui and Kanehara, 2013). Here, we showed that Rad9, which mediates Mec1-dependent Rad53 activation (Gilbert et al., 2001; Schwartz et al., 2002; Sweeney et al., 2005), is a key regulator for this masking. Rad9 cannot bind to Spo11-mediated DSBs in meiosis, whereas it does bind to HO-induced DSBs in mitosis. Indeed, tethering of Rad9 to meiotic DSBs by Ddc2-fusion induces the activation of Rad53 kinase.

**FIGURE 6:**
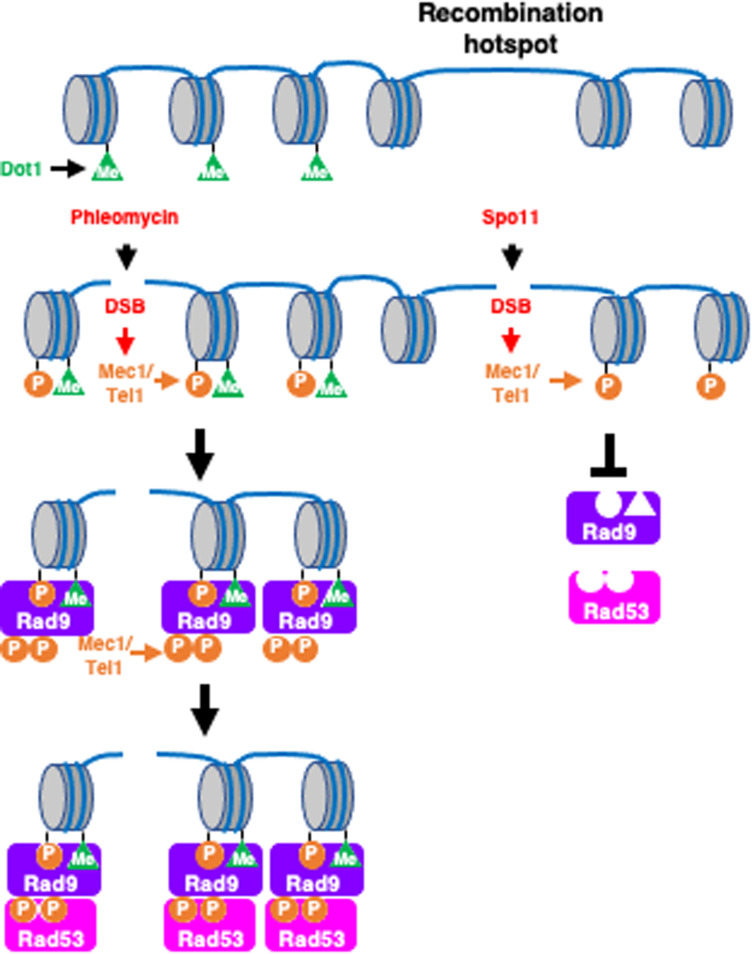
Rad9 is incompetent to meiotic DSBs. Exogenous DSBs often occur in nucleosome-rich gene body where Dot1-mediated histone H3K79 methylation marks are enriched (left pathway). The DSBs activates Mec1/Tel1, which in turn phosphorylates histone H2AS129. The two epigenetics marks, H3K79 methylation and H129S phosphorylation, provides multiple binding sites to Rad9. Rad9 is phosphorylated by Mec1/Tel1 and activates Rad53 kinase. At recombination hotspots is located in nucleosome-free region, which shows less H3K79 methylation compared to gene bodies right pathway. This chromatin features make the region insensitive to Rad9 binding.

The inability of Rad9 to bind to meiotic DSB may result from meiosis-specific modulation of Rad9 in order to inhibit the activity, either by posttranslational modification or through interaction with a meiosis-specific inhibitor. However, this latter possibility is less likely, given that Ddc2-Rad9 can activate Rad53 in response to meiotic programmed DSBs. If meiotic cells impose inhibition on Rad9 directly, Ddc2-Rad9 would also be inhibited in meiosis. Alternatively, in contrast to exogenous DSBs as well as HO-mediated DSBs, meiotic DSBs are intrinsically inert to Rad9 binding. Rad9 binding to chromosomes is promoted by 2 epigenetic markers in mitosis, histone H2AS129 phosphorylation and H3K79 methylation, which are recognized by 2 domains of Rad9, that is, BRCT and Tudor, respectively (Figure 6). H2AS129 phosphorylation is DNA damage-dependent and is mediated by Mec1/Tel1 checkpoint kinases, whereas H3K79 methylation is constitutive and is catalyzed by Dot1 methyltransferase. H2AS129 phosphorylation is induced by Spo11-mediated DSBs in meiosis in a Mec1-dependent manner (Eichinger and Jentsch, 2010). Interestingly, recent genome-wide mapping of H3K79 methylation showed that promoter regions of the yeast genome, where most of the meiotic DSBs occur (Buhler et al., 2007; Pan et al., 2011), tend to exhibit reduced levels of this modification (Zhang et al., 2011). One likely possibility is that this reduction of H3K79 methylation around DSB sites may partially explain the weak binding of Rad9 to meiotic DSBs (Figure 6). Consistent with this hypothesis, Rad53 activation by artificial targeting of Rad9 to the DSBs is independent of Dot1 or H3K79 methylation. However, previous studies have shown that meiotic cell cycle arrest in the *zip1* mutant, which is defective in chromosome synapsis and meiotic recombination, requires Dot1-dependent H3K79 methylation, suggesting that the methylation plays a role in response to meiotic DSBs, at least in the *zip1* mutant (Ontoso et al., 2013). We propose a simple model in which meiosis-specific chromosome structures play a role in making meiotic DSBs insensitive to Rad9, and thus to Rad53. This model is supported by a previous study that showed that Rad53 was activated when DSBs escaped from the recombination checkpoint and were present in meiosis-II, where meiosis-specific chromosome structures are dismantled (Cartagena-Lirola et al., 2008).

Why do meiotic cells mask Rad53 activation in response to Spo11-mediated DSBs? In this study, we found that the untimely activation of Rad53 kinase during meiosis has a negative impact on meiotic chromosome metabolism. The Ddc2-Rad9 fusion delayed meiotic repair of DSBs, suggesting that Rad53 activation is inhibitory to meiotic DSB repair, and thus meiotic recombination. Meiotic recombination preferentially occurs between homologous chromosomes (Schwacha and Kleckner, 1997). On the other hand, intersister recombination is preferred in mitotic recombination (Kadyk and Hartwell, 1992; Bzymek et al., 2010). One simple idea is that Rad53 activation inhibits interhomolog recombination or activates intersister recombination. However, this is less likely since *DDC2-RAD9* cells, in which Rad53 is activated, still show normal levels of crossover formation in meiosis (Figures 5E,F).

Although Rad53 activation was inhibited in wild-type meiosis, we did not observe drastic defects in artificial activation of the kinase by Ddc2-Rad9. The effect of Rad53 activation by Ddc2-Rad9 on meiotic events was weak. This finding might be attributable to the fusion protein restricting the activation of Rad53 very close to the DSBs. Rad9-Rad53 is generally more dynamic in nuclei for signaling, at least in mitotic cells with exogenous DSBs (Melo et al., 2001; Usui et al., 2009).

There are several downstream targets of the Rad53 pathway, including Dbf4-dependent Cdc7 kinase (DDK) (Lopez-Mosqueda et al., 2010; Zegerman and Diffley, 2010). In meiosis, DDK plays multiple roles such as in the formation of DSBs, expression of middle pachytene genes, monopolar spindle attachment, and efficient cleavage of meiosis-specific cohesion (Lo et al., 2008; Matos et al., 2008; Sasanuma et al., 2008; Katis et al., 2010). Thus, it is likely that, in meiosis, DDK should be constitutively activated during meiotic prophase-I by avoiding inhibitory Rad53 activation. Moreover, a recent study clearly showed that meiotic prophase develops a program to guarantee a longer prophase (G2) compared to the mitotic phase (Okaz et al., 2012). It is also possible that DSB-dependent Rad53 activation might inhibit the operation of this program in a timely manner. We speculate that meiotic cells ensure a longer prophase (G2) through the inactivation of the Rad53 checkpoint kinase.

In mitotic cells, in addition to Rad9, the other DDR mediator protein, Dpb11, plays a role for efficient activation of Rad53 (Pfander and Diffley, 2011; Puddu et al., 2011; di Cicco et al., 2017). The recruitment of Dpb11 to DSB sites is dependent of the phosphorylation of a C-terminal tail of Ddc1 in the Rad17-Ddc1-Mec3 checkpoint clamp (Rad9-Rad1-Hus1 in other organisms). Then, Dpb11 recruits Rad9 through CDK-dependent phosphorylation of Rad9. We have known little about the regulation of Dpb11 during meiosis. However, the involvement of the checkpoint clamp in meiotic DNA damage response is controversial (Shinohara et al., 2003; Shinohara et al., 2015).

In mammals, 53BP1, a Rad9 homolog, is recruited to DSB sites through multiple interactions, including various histone modifications (Huyen et al., 2004; Botuyan et al., 2006; Mailand et al., 2007; Stewart et al., 2009; Fradet-Turcotte et al., 2013). These interactions function in the positive regulation of 53BP1 recruitment. The present study suggests that negative control of Rad9 to the DSB is also a key process for modulating the DNA damage response under different physiological conditions.

## Data Availability Statement

The original contributions generated for this study are included in the article/Supplementary Material, further inquiries can be directed to the corresponding author.

## Author Contributions

TU and AS conceived and designed the experiments and prepared the manuscript. TU performed the experiments. Both authors contributed to the article and approved the submitted version.

## Conflict of Interest

The authors declare that the research was conducted in the absence of any commercial or financial relationships that could be construed as a potential conflict of interest.

## Supplementary Material

Supplementary Figure 1 H3K79me is required for Rad9-dependent Rad53 activation in meiosis after phleomycin treatment. **(A)** TCA-precipitated cell extracts from meiotic cells were prepared from wild-type (USY543/544), *dot1* (USY526/527), and *h3-K79R* (USY693/677) diploid strains, and were analyzed by western blotting using anti-Rad9, anti-Flag (for Rad53), anti-Histone H3K79- tri-methyl and anti-tubulin antibodies. “^32^P-Rad53” represented ^32^P-incorporation to Rad53 in the ISA assay. Cell extracts derived from 4 × 10^6^ cells were loaded for the ISA assay. Cell extracts were prepared at indicated time points after the treatment of 3.5-h meiotic cells with 5 μg/mL of phleomycin. **(B)** Diploid cells of the indicated strains were arrested at G2/M by nocodazole and treated with 5 μg/mL of phleomycin for 30 min, followed by TCA precipitation. Wild-type (USY543/544), *rad9* (USY524/525), *dot1* (USY526/527), and *dot1 rad9* (USY522/523) were used in the experiment.

Supplementary Figure 2 Ddc2-Rad9 fusions activate Rad53 in meiosis. **(A)** Cells in meiotic time course at indicated times were collected and TCA-precipitated meiotic cell extracts were analyzed by western blotting using anti-Flag, and anti–α-tubulin antibodies. Wild-type (USY543/544), *DDC2-RAD9* (USY544/661), *DDC2-rad9-7A* (USY544/667), and *DDC2-RAD9 rad53-KD* (USY545/720) were examined. **(B)** Cell extracts were prepared from the strains and analyzed by western blotting using anti-HA, anti-Flag and anti–α-tubulin antibodies; *DDC2-RAD9* (USY544/661), *DDC2-RAD9 rad53-KD* (USY545/720), 3x HA-tagged Rad9 (USY544/661; *OE-HA-RAD9*) from the *DMC1* promoter, and *DDC2-RAD9 spo11 rad53-KD* (USY545/720). **(C)** Cell extracts were prepared from the strains and analyzed by western blotting using anti-HA, anti-Flag, anti-histone H3K79-tri-methyl and anti–α-tubulin antibodies; Wild-type (USY543/544), *DDC2-RAD9* (USY544/661), *DDC2-rad9-Y798A* (USY544/776), *DDC2-RAD9 dot1* (USY768/526), and *DDC2-RAD9 h3-K79R* (USY770/693). **(D)** Cell extracts at 0, 3, and 4 h time points were prepared from the strains and analyzed by western blotting using anti-HA, anti-Flag, and anti–α-tubulin antibodies and also by ISA (third panel); Wild-type (USY543/544), *DDC2-RAD9* (USY544/661) and *DDC2-rad9-7A* (USY510/512). Cell extracts derived from 4 × 10^6^ cells were loaded for the ISA assay.

Supplementary Figure 3 Ddc2-Rad9 fusions activate Rad53 in *dmc1* meiosis. **(A)** Cell extracts at 0, 3, and 4.5 h time points of meiosis were prepared from the *dmc1*strains and analyzed by western blotting using anti-Flag and by ISA assay. Results from two independent time course are shown. Cell extracts derived from 4 × 10^6^ cells were loaded for the ISA assay. HA-Rad9 and Ddc1-HA-Rad9 protein were expressed from the *DMC1* promoter. *HA-RAD9 dmc1* (USY528/529), *DDC2-RAD9 dmc1* (USY528/529), and *DDC2-RAD9 dmc1 rad53-KD* (USY508/509). **(B)** Cell extracts at 0, 3, and 4.5 h time points of meiosis of the *dmc1* mutant were prepared from the strains and analyzed by western blotting using anti-HA and anti-Flag, and by ISA assay. *HA-RAD9 dmc1* (USY528/529), *DDC2-RAD9 dmc1* (USY528/529), and *DDC2-RAD9 dmc1 mec1* (USY458/459). **(C,D)** Cell extracts at 0, 1.5, 3, and 4.5 h time points of meiosis of the *dmc1* mutant were prepared from the strains and analyzed by western blotting using anti-HA and anti-Flag, and by ISA assay. **(C)** *DDC2-RAD9 dmc1* (USY528/529), and *DDC2-RAD9 dmc1 mec1* (USY458/459) and *DDC2-RAD9 dmc1 spo11* (USY514/515); **(D)** *HA-RAD9 dmc1* (USY528/529), *DDC2-RAD9 dmc1* (USY528/529), and *DDC2-RAD9 dmc1 rad53-KD* (USY508/509).

Supplementary Figure 4 TCA-precipitated extracts prepared from cells expressing Ddc2-3xHA (USY8384) or 3xHA-Rad9 (USY2035) from their native promoter and the indicated dilutions of *DDC2-RAD9* (USY544/671) and *HA-RAD9-OE* (USY544/561) extracts were examined. All cell extracts were prepared from 4 h meiotic cells. Cell extracts derived from 1 × 10^7^ or 4 × 10^6^ cells were loaded for anti-HA or anti-Flag/tubulin Western blot, respectively. Dilutions were prepared accordingly.

Supplementary Figure 5 The representative images of immunostaining of nuclear spreads prepared from *dmc1 RAD53 DDC2-RAD9* (USY580/591) at 6 h and *dmc1 rad53-KD DDC2-RAD9* (USY582/623) at 4.5 h in meiosis are shown with anti-HA and anti-Rad51. DNA images stained with DAPI are also presented. Bar equals 5 μm.

Supplementary Figure 6 **(A)** DNA contents of wild type (USY543/544) and *DDC2-RAD9* (USY544/671) strains were examined by FACS analysis. The representative profiles were shown. **(B)** The percentage of nuclei of the indicated strains that had more than five Rad51 or Dmc1 foci was plotted each time point.

## Abbreviations

ChIP: chromatin immuno-precipitation
DAPI: 4 ′, 6-diamidino-2-phenylindole
DSBs: DNA double-strand breaks
ISA: *in situ* autophosphorylation
SC: synaptonemal complex
WCE: whole cell extract.
