# Supplementary material for: Rad9, a 53BP1 Ortholog of Budding Yeast, Is Insensitive to Spo11-Induced Double-Strand Breaks During Meiosis
Source: Front Cell Dev Biol. 2021 Mar 25;9:635383. doi: 10.3389/fcell.2021.635383 (PMC8027355; doi:10.3389/fcell.2021.635383)
Supplement: Supplementary file 1 [file Data_Sheet_2.PDF]

Table S1: Strain list

Strain Name	Genotype
NKY1551	*MAT**a**/α, ho::LYS2/", lys2/", leu2::hisG/", ura3/", his4B-LEU2/his4X-LEU2-URA3, arg4-bgl/arg4-nsp*
USY20/USY35	*MAT**a**/α, ho::LYS2/", lys2/", leu2::hisG/", ura3/", his4B-LEU2/his4X-LEU2-URA3, arg4-bgl/arg4-nsp, HA-RAD9/"*
USY83/USY84	*MAT**a**/α, ho::LYS2/", lys2/", leu2::hisG/", ura3/", his4B-LEU2/his4X-LEU2-URA3, arg4-bgl/arg4-nsp, RAD9-3HA/"*
USY495/USY785	*MAT**a**/α, ho::LYS2/", lys2/", leu2::hisG/", ura3/", his4B-LEU2/his4X-LEU2-URA3, arg4-bgl/arg4-nsp , FLAG-RAD53/", DMC1::DMC1p-DDC2-HA-RAD9/DMC1, mec1:LEU2/", sml1X/"*
USY522/USY523	*MAT**a**/α, ho::LYS2/", lys2/", leu2::hisG/", ura3/", his4B-LEU2/his4X-LEU2-URA3, arg4-bgl/arg4-nsp, FLAG-RAD53/", rad9::HygMX6/", dot1::ZeoMX6/"*
USY524/USY525	*MAT**a**/α, ho::LYS2/", lys2/", leu2::hisG/", ura3/", his4B-LEU2/his4X-LEU2-URA3, arg4-bgl/arg4-nsp, FLAG-RAD53/", rad9::HygMX6/"*
USY526/USY527	*MAT**a**/α, ho::LYS2/", lys2/", leu2::hisG/", ura3/", his4B-LEU2/his4X-LEU2-URA3, arg4-bgl/arg4-nsp, FLAG-RAD53/", dot1::HygMX6/"*
USY543/USY544	*MAT**a**/α, ho::LYS2/", lys2/", leu2::hisG/", ura3/", his4B-LEU2/his4X-LEU2-URA3, arg4-bgl/arg4-nsp, FLAG-RAD53/"*
USY544/USY661	*MAT**a**/α, ho::LYS2/", lys2/", leu2::hisG/", ura3/", his4B-LEU2/his4X-LEU2-URA3, arg4-bgl/arg4-nsp , FLAG-RAD53/", DMC1::DMC1p-HA-RAD9/DMC1*
USY544/USY667	*MAT**a**/α, ho::LYS2/", lys2/", leu2::hisG/", ura3/", his4B-LEU2/his4X-LEU2-URA3, arg4-bgl/arg4-nsp, FLAG-RAD53/", DMC1::DMC1p-DDC2-HA-RAD9-7A/DMC1*
USY544/USY671	*MAT**a**/α, ho::LYS2/", lys2/", leu2::hisG/", ura3/", his4B-LEU2/his4X-LEU2-URA3, arg4-bgl/arg4-nsp , FLAG-RAD53/", DMC1::DMC1p-DDC2-HA-RAD9/DMC1*
USY544/USY767	*MAT**a**/α, ho::LYS2/", lys2/", leu2::hisG/", ura3/", his4B-LEU2/his4X-LEU2-URA3, arg4-bgl/arg4-nsp, FLAG-RAD53/", DMC1::DMC1p-DDC2-HA-RAD9-Y798A/DMC1*

USY544/USY797	*MAT**a**/α, ho::LYS2/", lys2/", leu2::hisG/", ura3/", his4B-LEU2/his4X-LEU2-URA3, arg4-bgl/arg4-nsp, FLAG-RAD53/", DMC1::DMC1p-DDC2-HA-RAD9-K1088M/DMC1*
USY545/USY720	*MAT**a**/α, ho::LYS2/", lys2/", leu2::hisG/", ura3/", his4B-LEU2/his4X-LEU2-URA3, arg4-bgl/arg4-nsp , FLAG-RAD53-KD/", DMC1::DMC1p-DDC2-HA-RAD9/DMC1*
USY559/USY666	*MAT**a**/α, ho::LYS2/", lys2/", leu2::hisG/", ura3/", his4B-LEU2/his4X-LEU2-URA3, arg4-bgl/arg4-nsp , FLAG-RAD53-KD/", DMC1::DMC1p-DDC2-HA-RAD9::dmc1::HygMX6/ dmc1::HygMX6, sp11::HygMX6/"*
USY580/USY591	*MAT**a**/α, ho::LYS2/", lys2/", leu2::hisG/", ura3/", his4B-LEU2/his4X-LEU2-URA3, arg4-bgl/arg4-nsp , FLAG-RAD53 /", DMC1::DMC1p-DDC2-HA-RAD9::dmc1::HygMX6/ dmc1::HygMX6*
USY582/USY623	*MAT**a**/α, ho::LYS2/", lys2/", leu2::hisG/", ura3/", his4B-LEU2/his4X-LEU2-URA3, arg4-bgl/arg4-nsp , FLAG-RAD53-KD/", DMC1::DMC1p-DDC2-HA-RAD9::dmc1::HygMX6/ dmc1::HygMX6*
USY582/USY674	*MAT**a**/α, ho::LYS2/", lys2/", leu2::hisG/", ura3/", his4B-LEU2/his4X-LEU2-URA3, arg4-bgl/arg4-nsp , FLAG-RAD53-KD/", DMC1::HA-RAD9::dmc1::HygMX6/ dmc1::HygMX6*
USY693/USY677	*MAT**a**/α, ho::LYS2/", lys2/", leu2::hisG/", ura3/", his4B-LEU2/his4X-LEU2-URA3, arg4-bgl/arg4-nsp, FLAG-RAD53/", rad9::HygMX6/", hht1-KK79R/", hht2-KK79R/"*
USY758/USY759	*MAT**a**/α, ho::LYS2/", lys2/", leu2::hisG/", ura3/", his4B-LEU2/his4X-LEU2-URA3, arg4-bgl/arg4-nsp, FLAG-RAD53/", rad9-Y798A:::URA3*
USY768/USY526	*MAT**a**/α, ho::LYS2/", lys2/", leu2::hisG/", ura3/", his4B-LEU2/his4X-LEU2-URA3, arg4-bgl/arg4-nsp , FLAG-RAD53/", DMC1::DMC1p-DDC2-HA-RAD9/DMC1, dot1::HygMX6/"*
USY770/USY693	*MAT**a**/α, ho::LYS2/", lys2/", leu2::hisG/", ura3/", his4B-LEU2/his4X-LEU2-URA3, arg4-bgl/arg4-nsp , FLAG-RAD53/", DMC1::DMC1p-DDC2-HA-RAD9/DMC1, hht1-*

	KK79R/", hht2-KK79R/"
USY783/USY414	*MAT**a**/*α, *ho::LYS2/"*, *lys2/"*, *leu2::hisG/"*, *ura3/"*, *his4B-LEU2/his4X-LEU2-URA3*, *arg4-bgl/arg4-nsp , FLAG-RAD53/", DMC1::DMC1p-DDC2-HA-RAD9/DMC1. Sp11-Y135F/"*
USY99	*MATa*, *FLAG-RAD53::LEU2*, *hoΔ hml1::ADE1*, *hmr::ADE1*, *ade1-100*, *leu2-3-112*, *trp1::hisG*, *lys5*, *ura3-52*, *ade3::GAL::HO bar1::ADE3::bar1*
USY100	USY99 but *HA-RAD9*

Table S2 Primer list

USP060_F-pFA-dmc1d
5’-
CCCTTTTATATTTGCTGTAGTCTGTTATGCCAATCAGGAAAGCATTTGAACAA
ATCCAGCTGAAGCTTCGTACGC
USP061_R-pFA-dmc1d
5’-
ATGTCATCTTCATTAAAAAAGTATACAAAAACTAGTCACTTGAATCGGTAATAC
CGCATAGGCCACTAGTGGATCTG

dot1Δ
YP180_F-pFA-dot1d
TCATCAAGGAGGTCACCAGTAATTGTGCGCTTTGGTTACATTTTGTTGTACA
GTACAGCTGAAGCTTCGTACGC
YP181_R-pFA-dot1d
GTTATTTCTACTTAGTTATTCATACTCATCGTTAAAAGCCGTTCAAAGTGCCT
CAGCATAGGCCACTAGTGGATCTG

USP114_F-pFA-rad9d
5’-
GATAGAGAAACGCCATAGAAAAGAGCATAGTGAGAAAATCTTCAACATCAG
GGCTCCAGCTGAAGCTTCGTACGC
USP010_R-pFA-Rad9-3UTR
TTTATTTAATCGTCCCTTTCTATCAATTATGAGTTTATATATTTTTATAATTTCAG
CATAGGCCACTAGTGGATCTG

USP107_F-pFA-spo11d
CTCACATATTTGTCTTCACCCTTAAGATTTTACGATTTACTAAGTTCACCTTCT
CCCAGCTGAAGCTTCGTACGC
USP108_R-pFA-spo11d
GTTTTCAATTCTTGAAAAACATTTTTTATAAAGCAACAGCTCCCATTCTTATTC
AGCATAGGCCACTAGTGGATCTG
