# Supplementary figures and images for: Rad9, a 53BP1 Ortholog of Budding Yeast, Is Insensitive to Spo11-Induced Double-Strand Breaks During Meiosis
Source: Front Cell Dev Biol. 2021 Mar 25;9:635383. doi: 10.3389/fcell.2021.635383 (PMC8027355; doi:10.3389/fcell.2021.635383)

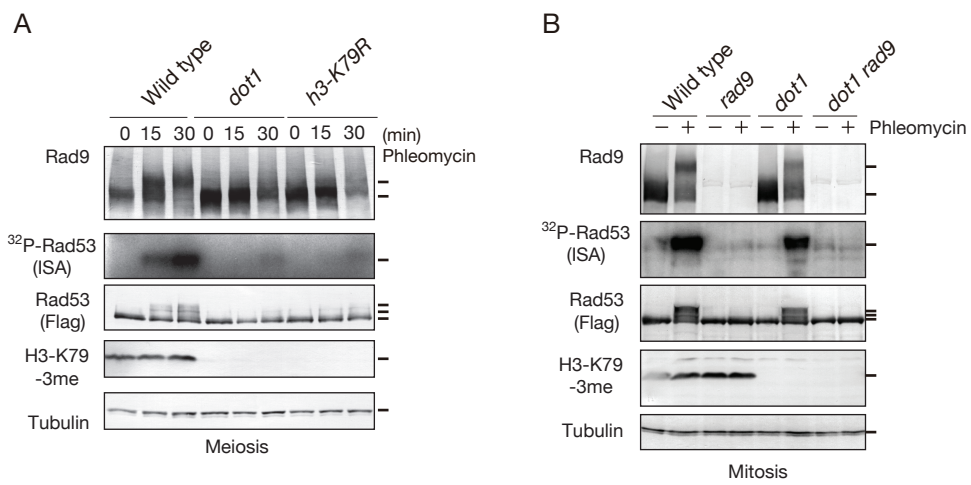

A

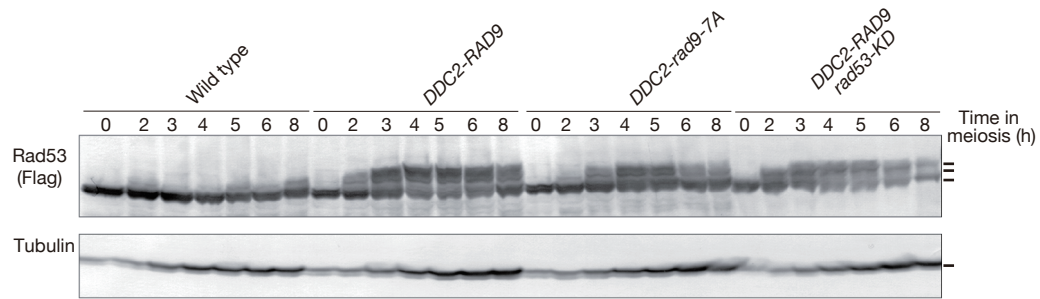

B

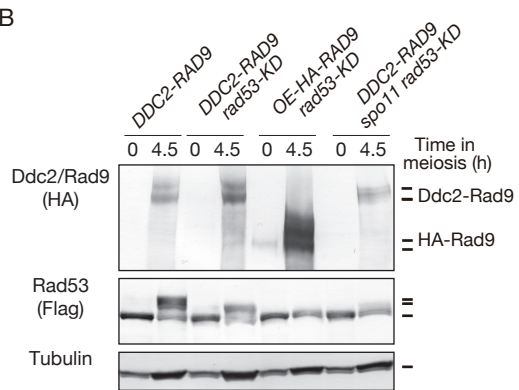

C

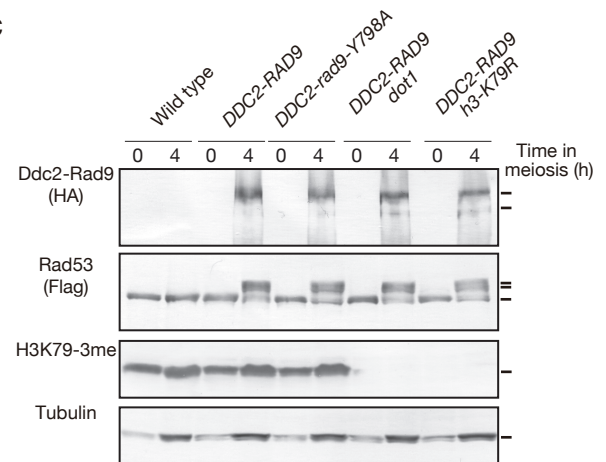

D

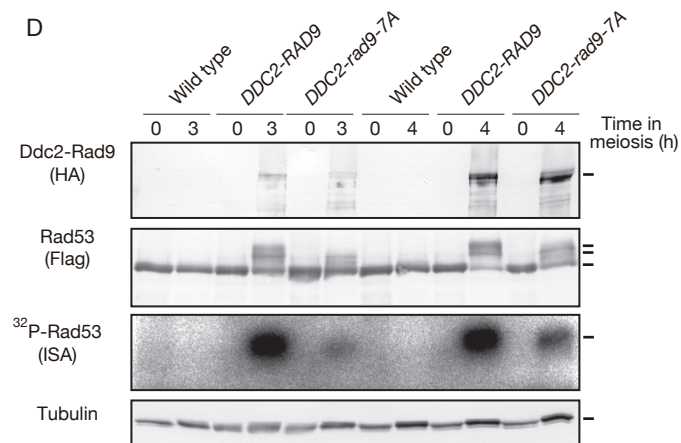

Figure S3 Usui et al.

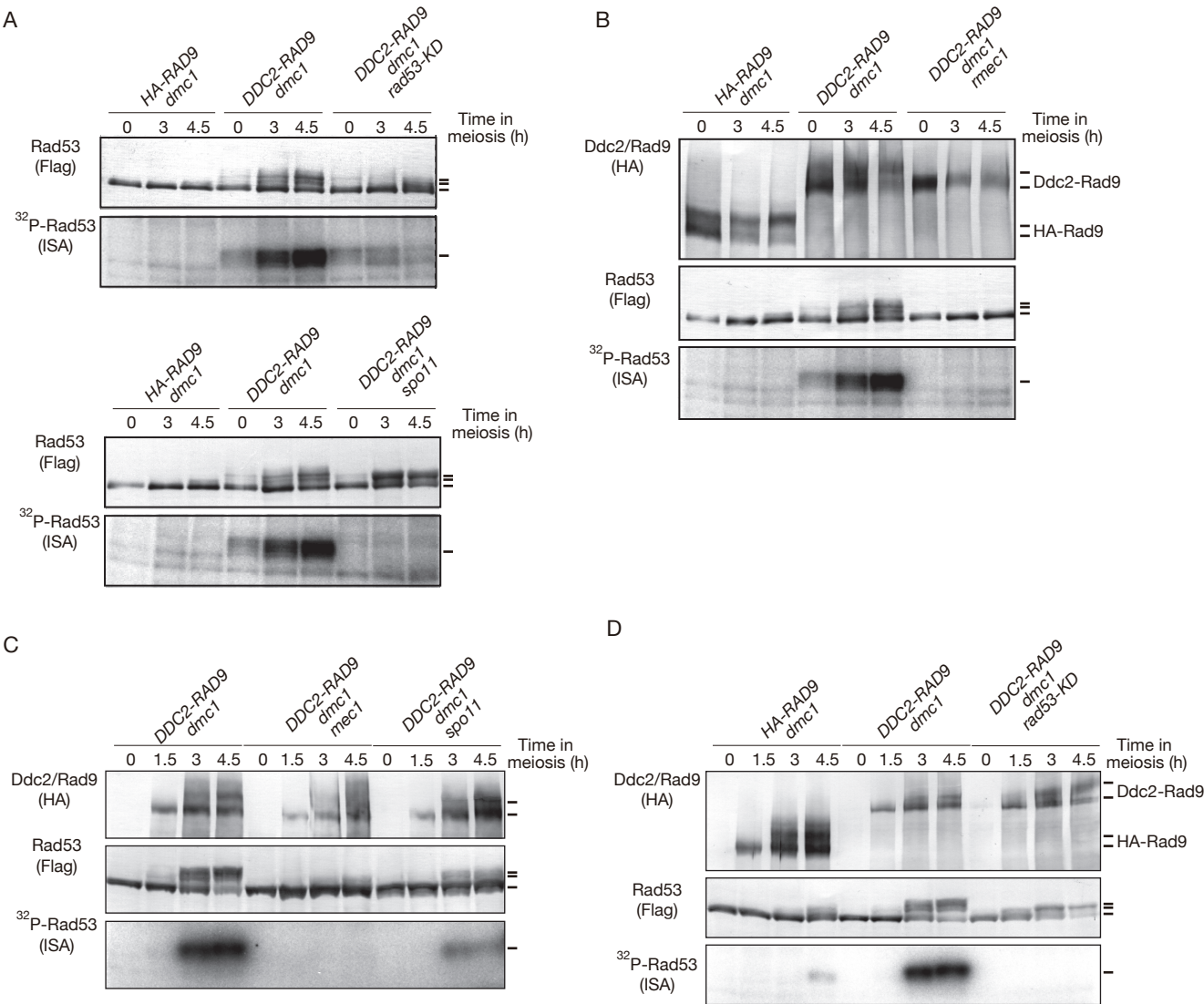

Figure S4 Usui et al.

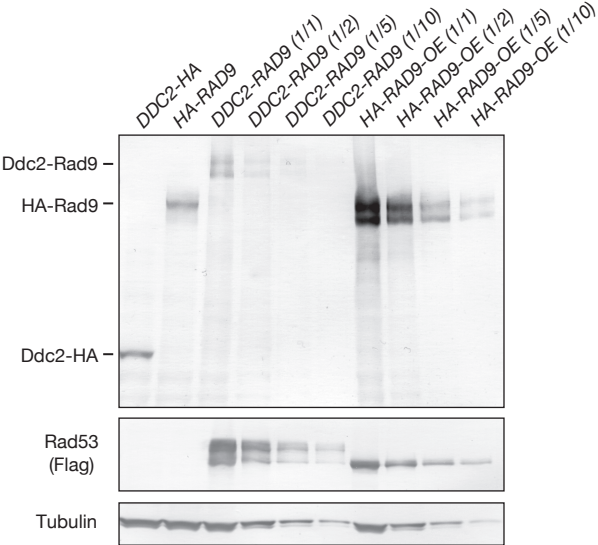

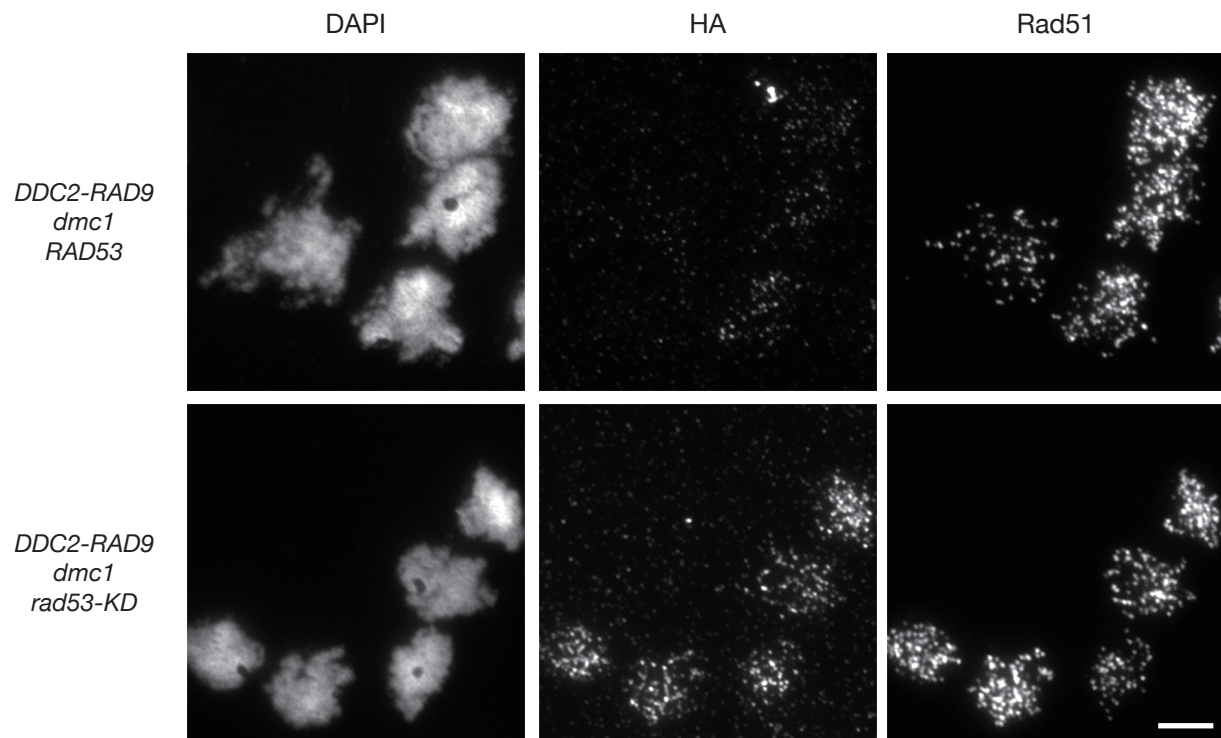

A

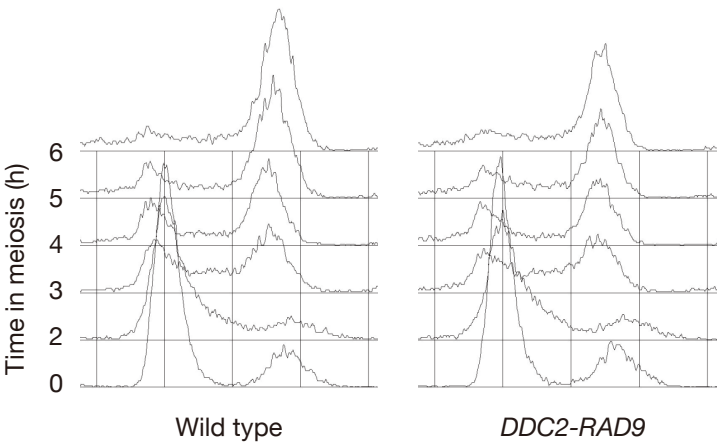

B

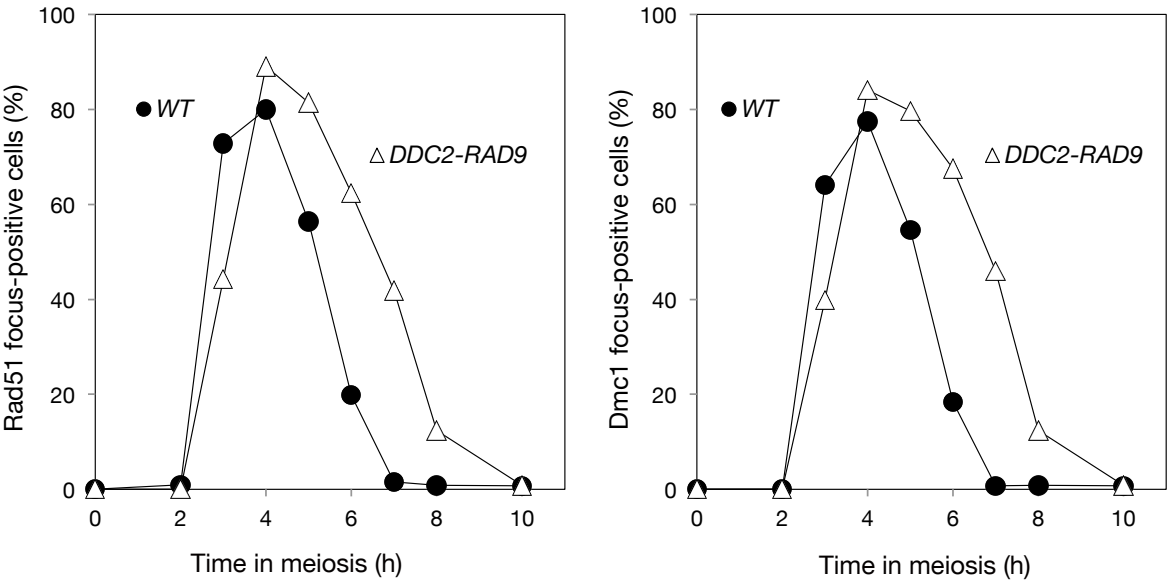

Supplement: Supplementary Figure 1 — H3K79me is required for Rad9-dependent Rad53 activation in meiosis after phleomycin treatment. (A) TCA-precipitated cell extracts from meiotic cells were prepared from wild-type (USY543/544), dot1 (USY526/527), and h3-K79R (USY693/677) diploid strains, and were analyzed by western blotting using anti-Rad9, anti-Flag (for Rad53), anti-Histone H3K79- tri-methyl and anti-tubulin antibodies. “32P-Rad53” represented 32P-incorporation to Rad53 in the ISA assay. Cell extracts derived from 4 × 10^6^ cells were loaded for the ISA assay. Cell extracts were prepared at indicated time points after the treatment of 3.5-h meiotic cells with 5 μg/mL of phleomycin. (B) Diploid cells of the indicated strains were arrested at G2/M by nocodazole and treated with 5 μg/mL of phleomycin for 30 min, followed by TCA precipitation. Wild-type (USY543/544), rad9 (USY524/525), dot1 (USY526/527), and dot1 rad9 (USY522/523) were used in the experiment. [file Data_Sheet_1.PDF]
